# Clinical validation of chemotherapy predictors developed on global microRNA expression in the NCI60 cell line panel tested in ovarian cancer

**DOI:** 10.1371/journal.pone.0174300

**Published:** 2017-03-23

**Authors:** Kira Philipsen Prahm, Claus Høgdall, Mona Aarenstrup Karlsen, Ib Jarle Christensen, Guy Wayne Novotny, Steen Knudsen, Anker Hansen, Peter Buhl Jensen, Thomas Jensen, Mansoor Raza Mirza, Anne Weng Ekmann-Gade, Lotte Nedergaard, Estrid Høgdall

**Affiliations:** 1 Department of Pathology, Molecular unit, Danish CancerBiobank, Herlev University Hospital, Herlev, Denmark; 2 Department of Gynecology, The Juliane Marie Centre, Rigshospitalet, Copenhagen University Hospital, Copenhagen, Denmark; 3 Medical Prognosis Institute, Hørsholm, Denmark; 4 Department of Oncology, Rigshospitalet, Copenhagen University Hospital, Copenhagen, Denmark; 5 Department of Pathology, Rigshospitalet, Copenhagen University Hospital, Copenhagen, Denmark; Seoul National University College of Pharmacy, REPUBLIC OF KOREA

## Abstract

**Objective:**

Ovarian cancer is the leading cause of death among gynecologic malignancies. This is partly due to a non-durable response to chemotherapy. Prediction of resistance to chemotherapy could be a key role in more personalized treatment. In the current study we aimed to examine if microRNA based predictors could predict resistance to chemotherapy in ovarian cancer, and to investigate if the predictors could be prognostic factors for progression free and overall survival.

**Methods:**

Predictors of chemotherapy-resistance were developed based on correlation between miRNA expression and differences in measured growth inhibition in a variety of human cancer cell lines in the presence of Carboplatin, Paclitaxel and Docetaxel. These predictors were then, retrospectively, blindly validated in a cohort of 170 epithelial ovarian cancer patients treated with Carboplatin and Paclitaxel or Docetaxel as first line treatment.

**Results:**

In a multivariate cox proportional analysis the predictors of chemotherapy-resistance were not able to predict time to progression after end of chemotherapy (hazard ratio: 0.64, 95% CI: 0.36–1.12, *P* = 0.117). However, in a multivariate logistic analysis, where time to progression was considered as either more or less than 6 months, the predictors match clinical observed chemotherapy-resistance (odds ratio: 0.19, 95% CI: 0.05–0.73, *P =* 0.015). Neither univariate nor multivariate, time-dependent, cox analysis for progression free survival (PFS) or overall survival (OS) in all 170 patients showed to match predicted resistance to chemotherapy (PFS: hazard ratio: 0.69, 95% CI: 0.40–1.19, *P =* 0.183, OS: hazard ratio: 0.76, 95% CI: 0.42–1.40, *P =* 0.386).

**Conclusion:**

In the current study, microRNA based predictors of chemotherapy-resistance did not demonstrate any convincing correlation to clinical observed chemotherapy-resistance, progression free survival, or overall survival, in patients with epithelial ovarian cancer. However the predictors did reflect relapse more or less than 6 months.

## Introduction

Ovarian cancer (OC) remains the most lethal gynecologic malignancy in the western world, and the 5^th^ most common cause of cancer death for women [1–3]. The majority of patients will be diagnosed in advanced stages (FIGO stage III-IV) where the 5-year overall survival rate is only 15–30% in Denmark [4]. This is primarily due to the late diagnosis, and despite improvements in combined chemotherapy; acquisition of resistance to chemotherapy is a major contributor to the low 5-year survival rate. Standard treatment of patients with OC is primary debulking surgery followed by adjuvant platinum-based combination chemotherapy [5]. Although the majority of patients initially respond well to chemotherapy, most of them will eventually experience relapses and eventually develops resistance to platinum based chemotherapy [4, 6–8]. Therefore there is an unmet need for biomarkers that can predict patients’ resistance to chemotherapy, spare patients from in-effective, toxic agents, and optimize treatment for each individual patient.

MicroRNAs (miRNAs) are small, 21–23 nucleotides long, non-coding RNA molecules that regulate gene expression by binding to the 3’-untranslated region of target genes that either induces mRNA degradation or represses translation of the protein [9, 10]. During the last decade it has been confirmed that miRNAs can function as tumor suppressors and oncogenes and play an important role in cancer [11]. Several studies have also identified miRNAs to be abnormally expressed in OC [12–15].

Currently there are no methods available for prediction of the individual patients’ resistance to chemotherapy, which is a key role in the development of personalized medicine. In this study we retrospectively validated miRNA based predictors, developed from the miRNA expression profile of a panel of cell lines that has been tested for their sensitivity to different chemotherapeutics. The aim of the study was to investigate if miRNA profiles can predict the sensitivity of platinum alone or the combined treatment of platinum and taxanes.

## Material and methods

### Patients and material

All patients for the current study were recruited from the Pelvic Mass study. The Pelvic Mass study was initiated in September 2004 at the Gynecologic Department, Rigshospitalet, Denmark. The study is a prospective ongoing study, with the intent to identify diagnostic and prognostic factors for OC. Patients with a potential malignant pelvic mass are invited to participate in the study, when admitted to Rigshospitalet for surgery. If radical surgery is considered possible a gynecologic oncologist operates the patient, and except for stage IA/IB low grade, all patients are subsequently offered chemotherapy. All histologic diagnoses are given by a pathologist specialized in gynecologic pathology, and the tissue is handled and stored by the Danish CancerBiobank [16]. Clinical information from each patient is registered online in the nationwide Danish Gynecological Cancer Database that covers information on more than 95% of all Danish patients diagnosed with ovarian, endometrial, vulva and cervical cancers [4]. The database further includes patient’s 10-digit national personal identification number, which enables linkage to other national registries. Information on death of any cause was obtained from the Civil Registration System [17].

Inclusion criteria were: OC with epithelial histology, primary surgery followed by treatment with a minimum of two cycles of adjuvant chemotherapy. Exclusion criteria were: Non epithelial OC, carcinosarcomas, neoadjuvant chemotherapy, no chemotherapy due to FIGO stadium IA, less than 2 series of adjuvant chemotherapy, patients who refrained from treatment, concomitant cancer disease, postoperative death or a poor medical condition that contraindicated treatment with chemotherapy, or insufficient tissue for analysis.

Progression Free Survival (PFS) was defined as the time from primary surgery until relapse, progressive disease (PD) or death of any cause which ever occurred first. Relapse and PD were defined from the best clinical evaluation, on the basis of CT/MRI/PET-CT scans, serum CA125 and patients’ symptoms. In 18 cases, where second line chemotherapy was initiated, but no information on relapse or PD was registered, start date of second line chemotherapy was considered as relapse or PD. Chemotherapy-resistance was defined as relapse or PD within six months after chemotherapy. If patients developed PD during treatment, or within 4 weeks after last cycle, they were considered chemotherapy-refractory. Patients were considered chemotherapy-sensitive if they had no relapse or PD, or if relapse or PD occurred more than 6 months after end of first line chemotherapy. Time from end of first line chemotherapy until relapse, PD or start of second line chemotherapy was designated time to progression and both the actual time, and time categorized as more, or less than 6 months, were used for statistical analyses of resistance to chemotherapy. Cause of death was defined as either, death of gynecologic cancer, or death of other causes. Patients were followed from date of surgery until death of any cause, emigration, or until January 17, 2015, which ever came first.

### Ethics statements

All patients included in the Pelvic Mass study are informed both in writing and orally and the commitment and participation is given with a written consent. The Danish Ethical Committee approves the Pelvic Mass protocol according to the rules of the International Conference on Harmonization/Good Clinical Practice (ICH/GCP) recommendations and the Helsinki and Tokyo conventions (KF01-227/03 and KF01-143/04).

### Microarray analysis

The miRNA analyses were made on formalin-fixed paraffin embedded (FFPE) tissue, where one slice of 20μm thickness from each patient was used. The tumor cell content was previously estimated from hematoxylin and eosin staining by a pathologist, specialized in gynecologic pathology, and was more than 50% in all of the tumors. miRNA was extracted using a total nuclei acid isolation kit for FFPE, RecoverAll (Ambion, Inc 2130 Woodward St. Austin, TX). miRNA was then labeled using FlashTag HSR^™^ Biotin RNA Labeling Kit (Genisphere, PA) and analyzed using GeneChip^®^ miRNA arrays (Affymetrix, CA).

### miRNA predictor developed on *in vitro* assay

To evaluate the correlation between miRNA expression and drug sensitivity, growth inhibition (GI50) vectors of the NCI60 cell line panel subjected to Carboplatin, Paclitaxel and Docetaxel were downloaded from the Developmental Therapeutics Program of the U.S National Cancer Institute’s web site. The NCI60 cell line panel consists of 60 different human cancer cell lines, including OC, and was developed as an *in-vitro* drug discovery tool for research in anti-cancer drug screening, and today function as a service screen tool for the cancer research community [18]. Correlation between miRNA expression and drug sensitivity in the cell lines was calculated for each miRNA–drug combination as previously described by Winther et al. [19]. miRNAs with a correlation above 0.25 (positively correlated miRNAs) or below -0.25 (negatively correlated miRNAs) were retained for each treatment, and then combined in order to predict sensitivity to combination treatment. Hence, three miRNA sensitivity profiles were developed in which miRNA expression levels were correlated to the sensitivity of the treatment regimens.

### Blind prediction of chemotherapy-resistance in clinical samples

The normalized expression of each miRNA in a sensitivity profile was used to predict sensitivity by turning the miRNA expression levels into a single prediction score. Hence, for each patient, sensitivity to the received treatment strategy was calculated as the difference between the average of positively correlated miRNAs and the average of negatively correlated miRNAs (prediction score = mean (positively correlated miRNAs)–mean (negatively correlated miRNAs)). Each miRNA in the profile was given equal weight. Next, the prediction score was normalized to a scale from 0 to 100 by a linear transformation of the prediction score of all patient samples. A score of zero meant least sensitive and a score of 100 most sensitive to the given treatment. For each patient, a score of predicted sensitivity to the received treatment strategy was calculated, while information of their clinical resistance to chemotherapy was blinded.

### Statistical analysis

The statistical analyses were performed according to an analysis plan included in the protocol finalized before the study.

Predictors of sensitivity for the drugs Carboplatin, Paclitaxel, Docetaxel, were combined and applied according to the treatment each patient had received. The sensitivity predictor was treated as a continuous variable and for statistical analysis divided by 50 resulting in odds ratios and hazard ratios for a 50 percent point difference in level.

For estimation of survival probabilities, Kaplan-Meier analysis was calculated by the tertiles of the predictor. The assumptions of proportionality and linearity were assessed with cumulated martingale residuals, and the assumptions were fulfilled. For estimation of median follow-up time, reverse Kaplan-Meier method was used.

Cox proportional hazards regression was used for both univariate and multivariate analysis of time to progression, progression free survival (PFS), and death (overall survival (OS)). Cancer specific survival was estimated with death of other causes as a competing risk [20].

Secondary univariate and multivariate analysis of chemotherapy-resistance was performed with logistic regression for time to progression categorized as more or less than 6 months. The multivariate analyses were adjusted for age, FIGO stage, histologic subtype and macroradical surgery. Preliminary analyses of interactions between relevant clinical parameters were performed.

Statistical significance was defined by a *P* value ≤ 0.05. The statistical analyses were performed using SAS statistical software packaged (version 9.4, Cary N.C. USA), R (v 3.1.0 R Development Core team, Vienna, Austria, http://www.R-project.org), and IBM SPSS statistical software version 19.

## Results

### Baseline characteristics

The first 246 patients consecutively included in the Pelvic Mass study, diagnosed with epithelial OC, were recruited for the study. 76 patients were excluded based on previously mentioned exclusion criteria, and were distributed as follows: Non epithelial OC (n = 2), Carcinosarcomas (n = 5), neoadjuvant chemotherapy (n = 15), no chemotherapy due to FIGO stadium IA (n = 8), less than 2 series of adjuvant chemotherapy (n = 3), patients who refrained from treatment (n = 4), concomitant cancer disease (n = 3), postoperative death (n = 5), a poor medical condition that contraindicated treatment with chemotherapy (n = 7), or insufficient tissue for analysis (n = 24). A total of 170 patients were eligible for inclusion in this study (S1 Clinical Data). There were no statistically significant difference between the 24 patients excluded due to insufficient tissue material, and the 170 patients included in the study according to age (p = 0.41), FIGO stage (p = 0.49) and histologic type (p = 0.19). For the patients included in the study, histologic diagnoses were as follows; 143 (83.6%) patients were diagnosed with serous carcinoma (low-grade: 3 (2%), high-grade: 140 (98%)), 10 (5.8%) patients with endometrioid carcinoma, 9 (5.3%) patients with clear cell carcinoma and 9 (5.3%) patients with mucinous carcinoma (Table 1). After primary debulking surgery, 81 (47.6%) patients had obtained macroradical surgery. Subsequently 165 (96.5%) patients received chemotherapy with the combination of Carboplatin and Docetaxel. 5 (2.9%) patients received single drug treatment with Carboplatin and one patient (0.6%) received treatment with the combination of Carboplatin and Paclitaxel.

**Table 1:** Baseline characteristics for 170 EOC patients.

Median age at diagnosis	63.9 (IQR: 54.2–72.5)
Median OS in months	51.1 (95% CI: 43.9–60.8)
Histologic type	
Serous adenocarcinoma	143 (84.1%)
Mucinous adenocarcinoma	9 (5.3%)
Edometrioid adenocarcinoma	9 (5.3%)
Clear Cell adenocarcinoma	9 (5.3%)
FIGO stage	
I	22 (12.9%)
II	18 (10.6%)
III	109 (64.1%)
IV	21 (12.4%)
Histologic grade^1^	
1	12 (7.1%)
2	95 (55.9%)
3	62 (36.5%)
Unknown^2^	1 (0.6%)
Residual tumor after surgery	
0 (macroradical surgery)	81 (47.6%)
< 1 cm	28 (16.5%)
> 1 cm ≤ 2 cm	20 (11.8%)
> 2 cm	41 (24.1%)
Time to progression	
> 6 months	124 (72.9%)
≤ 6 months	26 (15.3%)
Chemotherapy-refractory	20 (11.8%)

^1^Grade 1 = well differentiated, Grade 2 = moderately differentiated, Grade 3 =

^2^The pathologists were not able to determine grade due to necrotic tissue destruction.

FIGO = International Federation of Gynecology and Obstetrics, OS = overall survival, IQR = interquartile range.

At end of follow up a total of 114 (67.1%) patients had died, and 56 patient were still alive (32.9%). 126 (74.1%) patients had experienced relapse or PD, and 44 (25.9%) patients were alive without relapse. Median follow-up time was 86.2 months (range: 61.1–127.4), and median OS was 51.1 months (95% CI: 43.9–60.8). Out of the 170 patients, 81 (47.6%) patients obtained macroradical surgery. Patients who were sensitive to chemotherapy amounted 124 (72.9%). Twenty-six patients (15.2%) were resistant to chemotherapy and twenty patients (11.8%) were considered chemotherapy-refractory (Table 1).

### Prediction of chemotherapy-resistance and survival

The miRNAs that demonstrated the best correlation with sensitivity were identified, and selected for use in development of a prediction score for each patient. The miRNAs used for the prediction are listed in Table 2.

**Table 2:** List of miRNAs used for prediction of response to chemotherapy.

	Positive	Negative
Drug		
**Carboplatin**	hsa-miR-124_st	hsa-miR-10a_st
	hsa-miR-143_st	hsa-miR-183_st
	hsa-miR-1271_st	hsa-miR-192-star_st
	hsa-miR-342-3p_st	hsa-miR-192_st
	hsa-miR-370_st	hsa-miR-194_st
	hsa-miR-433_st	hsa-miR-200a-star_st
	hsa-miR-654-3p_st	hsa-miR-200a_st
	hsa-miR-758_st	hsa-miR-200b-star_st
	U55_x_st	hsa-miR-200b_st
		hsa-miR-200c-star_st
		hsa-miR-203_st
		hsa-miR-29b_st
		hsa-miR-30b_st
		hsa-miR-30d_st
		hsa-miR-429_st
		hsa-miR-625_st
		hsa-miR-7_st
**Paclitaxel**	hsa-miR-106b-star_st	**HBII-85-29_st**
	hsa-miR-1228_st	hsa-let-7e_st
	hsa-miR-185_st	hsa-miR-125a-5p_st
	hsa-miR-188-5p_st	hsa-miR-130a_st
	hsa-miR-18b_st	hsa-miR-193b_st
	hsa-miR-20b_st	hsa-miR-22_st
	hsa-miR-25_st	hsa-miR-27a_st
	hsa-miR-320c_st	**hsa-miR-29a_st**
	hsa-miR-320d_st	**hsa-miR-29b_st**
	hsa-miR-362-5p_st	hsa-miR-30a-star_st
	hsa-miR-500-star_st	hsa-miR-30a_st
	hsa-miR-500_st	hsa-miR-30c-2-star_st
	hsa-miR-501-3p_st	hsa-miR-30c_st
	hsa-miR-502-3p_st	**hsa-miR-34a_st**
	hsa-miR-532-3p_st	hsa-miR-34b-star_st
	hsa-miR-532-5p_st	**hsa-miR-34c-3p_st**
	hsa-miR-652_st	**hsa-miR-34c-5p_st**
	hsa-miR-766_st	
**Docetaxel**	hsa-miR-1307_st	HBII-438A_s_st
	hsa-miR-505_st	HBII-85-11_st
	hsa-miR-769-3p_st	HBII-85-15_x_st
	hsa-miR-769-5p_st	HBII-85-23_x_st
		**HBII-85-29_st**
		HBII-85-29_x_st
		hsa-miR-184_st
		**hsa-miR-29a_st**
		**hsa-miR-29b_st**
		**hsa-miR-34a_st**
		**hsa-miR-34c-3p_st**
		**hsa-miR-34c-5p_st**
		hsa-miR-424-star_st

Both positively and negatively miRNAs that were correlated to drug sensitivity of each drug are presented.

Positive = correlation above 0.25. Negative = correlation below -0.25.

MiRNAs marked in bold represent the miRNAs that are identified for both Paclitaxel and Docetaxel.

The primary univariate and multivariate cox proportional regression analyses, of the progression scores modelling time to progression, did not demonstrate a significant association (Univariate hazard ratio (HR): 0.99, 95% CI: 0.62–1.59, *p =* 0.97. Multivariate HR: 0.64, 95% CI: 0.36–1.12, *p* = 0.117), Table 3.

**Table 3:** Multivariate cox analyses of the miRNA prediction score modelling time to progression from end of last chemotherapy (n = 170).

	HR	95% CI	*P*-value
Prediction score	0.64	0.36–1.12	0.117
Age	1.11	0.94–1.31	0.204
FIGO stage			
I	0.09	0.02–0.34	**0.0004**
II	0.53	0.22–1.28	0.159
III	0.94	0.53–1.66	0.823
IV	-	-	-
Histologic type			
Serous adenocarcinoma	0.55	0.19–1.61	0.276
Mucinous adenocarcinoma	0.48	0.11–2.09	0.325
Endometriod adenocarcinoma	0.15	0.03–0.89	**0.037**
Clear cell adenocarcinoma	-	-	-
Macroradical surgery (yes/no)	0.32	0.21–0.49	**<0.001**

HR = hazard ratio, 95% CI = 95% confidence interval, FIGO = International Federation of Gynecology and Obstetrics.

Bold *p-*values indicate a significant result.

The secondary univariate logistic regression analysis in prediction of chemotherapy-resistance was not significant (OR 0.47 (95% CI: 0.17–1.30), AUC = 60%, *p* = 0.15). However the multivariate logistic regression analysis, adjusted for age, FIGO stage, histologic subtype and macroradical surgery, showed a significant correlation between the prediction score and chemotherapy-resistance more or less than 6 months (OR 0.19 (95% CI: 0.05–0.73) *p* = 0.0152), Table 4.

**Table 4:** Multivariate logistic regression analysis of the miRNA prediction score modelling chemotherapy-resistance (defined as progression/relapse > 6 months, in the clinical cohort (n = 170).

	OR	95% CI	*P*-value
Prediction score	0.19	0.05–0.73	**0.0152**
Age	1.01	0.70–1.47	0.949
FIGO stage			
I	-	-	-
II	1	-	-
III	1.64	0.29–9.43	0.576
IV	4.73	0.61–36.36	0.136
Histologic type			
Serous adenocarcinoma	1	-	-
Mucinous adenocarcinoma	0.37	0.03–4.66	0.441
Endometriod adenocarcinoma	2.45	0.26–23.18	0.435
Clear cell adenocarcinoma	12.61	1.27–125.03	**0.0303**
Macroradical surgery (yes/no)	7.16	2.40–21.35	**0.0004**

OR = odds ratio, 95%

CI = 95% confidence interval

FIGO = International Federation of Gynecology and Obstetrics.

Bold *p-*values indicate a significant result.

Univariate cox proportional hazards model for survival was not statistically significant (PFS: HR 1.0 (95% CI: 0.62–1.60) p = 0.10; OS: HR 0.97 (95% CI 0.58–1.61) p = 0.90), cancer specific survival (p = 0.76)). Multivariate cox regression analyses for prediction of survival showed a trend towards longer survival for higher values of the prediction score, but no significant association was demonstrated (PFS: HR 0.69 (95% CI: 0.40–1.19) *p* = 0.183; OS: 0.76 (95% CI: 0.42–1.40) *p* = 0.386), Table 5.

**Table 5:** Multivariate, cox analysis of the miRNA prediction score, predicting survival in the clinical cohort (n = 170).

	HR	95% CI	*P*-value
**PFS**			
Prediction score	0.69	0.40–1.19	0.183
Age	1.08	0.91–1.27	0.391
FIGO stage			
I	0.09	0.02–0.34	**0.0004**
II	0.63	0.26–1.53	0.308
III	0.98	0.56–1.72	0.943
IV	1	-	-
Histologic type			
Serous adenocarcinoma	1	-	-
Mucinous adenocarcinoma	0.94	0.33–2.72	0.913
Endometriod adenocarcinoma	0.28	0.07–1.18	0.082
Clear cell adenocarcinoma	1.86	0.63–5.52	0.262
Macroradical surgery	3.49	2.23–5.45	**<0.0001**
**OS**			
Prediction score	0.76	0.42–1.40	0.386
Age	1.23	1.03–1.47	**0.0202**
FIGO stage			
I	1	-	-
II	3.67	1.04–12.92	**0.0431**
III	7.41	2.46–22.36	**0.0004**
IV	8.36	2.44–28.66	**0.0007**
Histologic type			
Serous adenocarcinoma	1	-	-
Mucinous adenocarcinoma	2.42	0.95–6.15	0.065
Endometriod adenocarcinoma	0.38	0.09–1.60	0.189
Clear cell carcinoma	2.43	0.82–7.18	0.108
Macroradical surgery	2.63	1.61–4.26	**0.0001**

HR = hazard ratio, 95%

CI = 95% confidence interval

PFS = progression free survival

OS = overall survival

FIGO = International Federation of Gynecology and Obstetrics.

Bold *p-*values indicate a significant result.

Inclusion of menopausal status in the multivariate analyses did not show to be significant for any of the endpoints time-to-progression from end of last chemotherapy, chemotherapy-resistance, PFS or OS, (p-values: 0.86, 0.77, 0.73, 0.87) and age showed to be more associated with outcome. Therefore menopausal status was not included in the multivariate analyses.

## Discussion

As the gynecologic cancer with the poorest prognosis, OC is an important disease where continuous research that could improve prognosis for the patients, remains an import goal. Despite most patient respond well to first line chemotherapy, the majority eventually develop resistance to the treatment. A rational approach to identify patients, who will respond to a given chemotherapy before initiation of the treatment, could aid in the more personalized medicine and potentially improve survival of the patients.

In the current study prediction scores of resistance to combinations of Carboplantin, Docetaxel and Paclitaxel were developed in 170 OC patients based on their tumor tissue miRNA expression, and the global miRNA expression and growth response of the NCI60 cell line panel. In the univariate and multivariate analyses of chemotherapy-resistance modelling time to progression, adjusted for relevant prognostic factors, the miRNA based predictors were insignificant predictors of chemotherapy-resistance.

In secondary analyses, when chemotherapy-resistance was defined as time to progression or death more or less than 6 months, the miRNA predictors showed an association with resistance in multivariate logistic analysis, adjusted for relevant clinical factors (*p* = 0.015).

For PFS and OS a trend towards higher values of the prediction scores were associated with longer PFS and OS, however the results were insignificant.

The current method used for development of the miRNA predictor, is based on a novel bioinformatic approach that has been described in two former, published studies [19, 21]. In the first study, miRNA predictors of sensitivity to CHOP (Cyclophosphamide, Doxorubicin, Vincristine and Prednisone) and CHOEP (Cyclophosphamide, Doxorubicin, Vincristine, Etoposide and Prednisone) were developed and blindly validated in a cohort of 116 patients with diffuse large B-cell lymphoma. The study demonstrated that the miRNA predictors were able to predict the patients sensitivity to CHOP and CHOEP [21]. The most recent study investigated if miRNA based predictors of sensitivity to Cisplatin, Epirubicine and Capecitabine were predictive of survival in patients with gastroesophageal cancer, and in both univariate and multivariate analyses they found the miRNA profiles, predictive for the chemotherapeutics, to be independently associated with overall and disease free survival [19].

Prediction of OC patients’ sensitivity to chemotherapy is an important factor for improvement of prognosis. Currently, there are no molecular methods that accurately can predict chemotherapy-sensitivity, and guide clinicians in the selection of the potentially most effective therapy for the individual patient with OC.

miRNAs have in previous studies shown to be possible biomarkers for prediction of sensitivity to chemotherapy. The most frequently reported miRNAs shown to be associated with chemotherapy-sensitivity are the let-7 and the miR-200 families [22–24]. Yang *et al*. found that let-7i was significantly deregulated in chemotherapy-resistant patients with EOC, illustrating the let-7 family tumor suppressor function that also has been demonstrated in others studies [22, 25, 26]. However, in another study, identifying miRNAs to be altered in human OC resistant cell lines, let-7e showed to be upregulated in Paclitaxel-resistant (A2780TAX) cells, but downregulated in other, both Paclitaxel- and Cisplatin-resistant, cell lines, whereas miR-30c was downregulated in all Paclitaxel- and Cisplatin-resistant cell lines. Also miR-130a showed to be downregulated in all resistant cell lines, and suggested to exert its effect by targeting M-CSF, known to enhance invasiveness and metastasis in OC [23]. In our study, let-7e, miR-30c and miR-130a were negatively correlated to Paclitaxel, but only one patient in our study was treated with Paclitaxel. The studies of the miR-200 family in association with drug-resistance in OC are conflicting. The miR-200 family is an important regulator of epithelial-to-mesenchymal transition (EMT) that has a central role in cancer cell invasion, migration and drug resistance in several types of cancers [27–32]. Leskela *et al*. showed that patients with low expression of miR-200c was associated with high b-tubulin III protein content and lack of complete response to Platinum/Taxane-based chemotherapy [33]. In resemblance, two other studies have demonstrated that upregulation of miR-200c sensitized OC cell lines to Carboplatin and Paclitaxel [27, 29]. In other studies, upregulation of miR-200a and miR-141 has been reported to restore sensitivity to Paclitaxel and Carboplatin [28, 34]. Furthermore, one study found that upregulation and introduction of mimics of the miR-200 family members in the paclitaxel resistant OVCAR-3/TP cells were unable to restore sensitivity to Paclitaxel and further increased resistance to Carboplantin, particularly miR-200c and miR-141 mimic. However, miR-200c and miR-141 mimics did sensitize MES-OV/TP cells to paclitaxel. They therefore concluded that restoration of sensitivity, by upregulation and mimics of the miR-200 family members, depended on cell context, as the different cell lines had different expression levels of the miR-200 family members [35]. In our study miR-200a, miR-200b and miR-200c showed to be negatively correlated to Carboplatin sensitivity. For Carboplatin alone, miR-370 has shown to promote chemo-sensitivity in endometrioid OC [36]. This is in line with our findings, where miR-370 showed to be positively correlated to Carboplatin sensitivity. In contrast to our findings, where miR-29b has shown to be negatively correlated to Carboplatin sensitivity, a previous study found overexpression of miR-29a/b/c to sensitize OC cells to Cisplatin [37]. The full understanding of miRNAs regulation of chemotherapy-resistance in OC needs much further research. A comparison of the miRNA function found in the current study and in the literature is given in Table 6. It was previously revealed in an analysis of the Cancer Genome Atlas that no clear single miRNA signature can predict chemotherapy-sensitivity in patients, addressing the multifactorial nature of drug resistance in OC [38]. Furthermore, it has been demonstrated that some of the ways miRNAs affect drugs resistance could only be demonstrated in *in vitro* models, which underscores the importance of using a variety of models for studying the roles of miRNAs [39]. In the current study we combined information of *in vitro* cell lines response to drugs, and microarray analyses on tumor tissue from OC patients combined with their clinical information of response to chemotherapy in order to develop a method for prediction of response to chemotherapeutics. However the conflicting results of the miRNAs regulation of chemotherapy-resistance found in previous studies, indicate that the function of the miRNAs are still unclear, and might not be specific enough for detection of resistance, but could also be addressed to the various analyses methods and designs used in the different studies.

**Table 6:** Comparison of miRNAs and chemotherapy response in the current study and the literature.

miRNA	Response shown in the current study	Response shown for OC in the literature
Let-7e	Negatively correlated to paclitaxel sensitivity	**↑↓** in paclitaxel and cisplatin resistant cells [23]
miR-30c	Negatively correlated to paclitaxel sensitivity	**↓** in cisplatin + paclitaxel resistant cells [23]
miR-130a	Negatively correlated to paclitaxel sensitivity	**↓** in cisplatin + paclitaxel resistant cells [23]
miR-200a	Negatively correlated to carboplatin sensitivity	↑ in carboplatin + paclitaxel sensitive cells [28] ↑ enhanced sensitivity to paclitaxel, but not cisplatin [40]
miR-200c	Negatively correlated to carboplatin sensitivity	↓ in platinum/taxane resistant cells [33] ↑ restored carboplatin and paclitaxel sensitivity [27, 29]
miR-429	Negatively correlated to carboplatin sensitivity	↑ increased sensitivity to cisplatin [41] ↑ in drug sensitive patients [42]
miR-370	Positively correlated to carboplatin sensitivity	**↑** promotes carboplatin sensitivity in endometroid OC [36]
miR-29b	Negatively correlated to carboplatin, paclitaxel and docetaxel sensitivity	↓ increased resistance to cisplatin [37] ↑ sensitized cells to paclitaxel [43]
miR-27b	Negatively correlated to paclitaxel sensitivity	**↑** in paclitaxel resistant cell lines [44]

Positive = correlation above 0.25.

Negative = correlation below -0.25.

The arrows symbolize either up-regulation (↑) or down-regulation (↓) of the miRNA.

To our best knowledge this is the first study to investigate a global miRNA predictor of chemotherapy-resistance in OC patients. Although the miRNA predictors were not independent predictors of chemotherapy-resistance or survival in the primary analyses, a trend was observed. However we did see that the predictors were significant predictors of chemotherapy-resistance, when resistance was categorized (progression or death more or less than 6 months). The analysis further showed that patients with residual tumor after primary surgery had increased risk of progression within the first 6 month after end of chemotherapy, as would be expected. Preliminary analysis of interaction between the miRNA predictors and marcroradical surgery were performed, and none were observed. Therefor subgroup analysis on patients with residual tumor was not relevant. Since only six OC cell lines are included in the NCI60 panel, it might be one of the reasons the predictors failed to show a general significant result. However, the reasons the miRNA predictors fail to predict OS may also be attributed to the term survivalpost progression, especially if survivalpost progression is long [45].

The strengths of the current study are the consecutively inclusion of patients, where clinical information is continuously updated in the Danish Gynaecologic Cancer Database the long follow-up time, where all patients have been followed for at least five years, and none were lost to follow-up [4].

The current study was a retrospective validation study of the miRNA-based predictors of chemotherapy-resistance. The relative small number of patients included in the study should be noted as a weakness, as well as the number of patients excluded from the current analysis, which potentially could have biased the interpretation of the current results, and would have rendered it difficult to apply the results to the general population. However, the group of excluded patients, due to insufficient tissue material, was comparable to the groups of included patients in the analysis according to age, FIGO stage, and histologic type. Further the cohort only included patients from a single center, and resistance was only evaluated on results from first-line chemotherapy, where patients have a high response rate.

Improvements in treatment of OC with better surgical techniques, new antiangiogenic drugs and PARP inhibitors has within recent years improved survival, so time to progression has been prolonged considerably [46–48]. However, the majority will eventually develop resistance, and responses to subsequent treatments are generally short-lived. Therefore prediction of the potentially most effective second line treatment for those patients who relapse would be very important. Unfortunately the current developed miRNA based predictors did not demonstrate to be clinical relevant predictors of chemotherapy-resistance or survival in patients with OC.

## Conclusion

In the current study miRNA based predictors of chemotherapy-resistance were not able to demonstrate significant associations with resistance to treatment with Carboplatin, Docetaxel and Paclitaxel in patients with OC. However in secondary analyses the predictors did reflect relapse more or less than 6 months after end of primary chemotherapy. Larger studies where subgroup analyses are possible are warranted.

## Supporting information

S1 Clinical Data (SAV)
